# Comparison of the Retention and Separation Selectivity of Aromatic Hydrocarbons with Polar Groups in RP-HPLC Systems with Different Stationary Phases and Eluents

**DOI:** 10.3390/molecules25215070

**Published:** 2020-11-01

**Authors:** Anna Klimek-Turek, Beata Misiołek, Tadeusz H. Dzido

**Affiliations:** 1 Department of Physical Chemistry, Medical University of Lublin, Chodźki 4a, 20-093 Lublin, Poland; beata.misiolek@umlub.pl (B.M.); tadeusz.dzido@umlub.pl (T.H.D.); 2 Department for Variations and Renewals of Medicinal Products, The Office for Registration of Medicinal Products, Medical Devices and Biocidal Products, Al. Jerozolimskie 181C, 02-222 Warsaw, Poland

**Keywords:** stationary phases, molecular interactions, separation selectivity, HPLC

## Abstract

In this manuscript, the retention of aromatic hydrocarbons with polar groups has been compared for systems with various nonpolar columns of the types from C3 to C18 and different mobile phases composed of methanol, acetonitrile, or tetrahydrofuran as modifiers. The selectivity separation of the solutes in systems with different adsorbents, when one eluent modifier is swapped by another, has been explained, taking into account molecular interactions of the solutes with components of the stationary phase region (i.e., extracted modifier depending on the chain length of the stationary phase).

## 1. Introduction

Reverse-phase high-performance liquid chromatography (RP-HPLC) is one of the most commonly used analytical methods applicable in the pharmaceutical [1,2,3] and cosmetic industries [4,5,6], food analysis [7,8,9], diagnostics [10,11], biomedicine [12], environmental protection [9], and many other fields [13]. The main advantage of HPLC as an analytical method is the possibility of using many options for changing the retention and separation selectivity [11,14,15], like selecting the polarity of the eluent [16], tailoring the mobile phase, and so forth [17,18,19]. However, some aspects of the chromatographic process, especially those related to retention and separation selectivity mechanisms, are still debatable [20,21,22,23,24,25,26,27]. It is widely accepted that retention could be caused by adsorption of the analytes onto the adsorbent surface [28] and/or partitioning of the analytes between the stationary and the mobile phases [29,30]. The properties of the stationary phase strongly depend on the qualitative and quantitative compositions of the mobile phase [31]. The components of the mobile phase may adsorb on the stationary phase, forming a layer or even many layers [32,33,34,35]. The type and amount of adsorbed molecules of organic modifier have a strong influence on retention and selectivity [36,37,38]. Therefore, the stationary phase cannot be considered only as hydrocarbon chains with a certain length but as a combination of four components: hydrocarbon chains, solvent molecules, water molecules, and residual silanols [39,40]. Therefore, it is very hard to interpret the retention changes and put forward a clear conclusion.

However, it is worth noting that nonpolar stationary phases of the C18 type of similar carbon density in systems with various organic modifiers are characterized by constant composition of the hydrocarbon chains, unreacted silanol groups, and water (in the limited range of modifier concentration) [39]. It means that the modifier in such chromatographic system is responsible for different stationary phase properties. In addition, the partition constant of aromatic hydrocarbons with different polar groups in gas–liquid systems, where liquid is composed of organic modifier (methanol (MeOH), acetonitrile (ACN), or tetrahydrofuran (THF)) in water, demonstrates a very good correlation [41]. Based on these circumstances, in our previous papers we proposed an approach in which separation selectivity can be explained by molecular interactions of solutes and the component of the stationary phase when one modifier is replaced by another [39,41]. The application of this approach to the explanation of separation selectivity changes was demonstrated, among others, for aliphatic hydrocarbons with a polar group [42] and aromatic hydrocarbons with different polar groups [43] in systems with C18 stationary phase type.

The results presented in our previous papers showed a clear dependence of the changes in separation selectivity on the mobile phase modifier type [39]. They also allowed us to develop rules explaining these changes. However, these rules refer to those stationary phases that were investigated so far (i.e., C18 type as mentioned above). In view of the wide range of stationary phases, it is justified to check whether the above-described approach is also applicable to systems with other nonpolar adsorbents containing alkyl chains of different lengths, and also differing in the degree of coverage with these chains, the size of the pores, and so forth. It is well known that the total amount of adsorbed organic solvent depends on the length of hydrocarbon chains [44], the density of the column surface coverage [44], and the presence of free silanol groups [45]. When the surface coverage and number of carbons in ligands increase, the amount of the adsorbed solvent also increases, but it is worth noting that when the coverage density is very high, molecules of an organic modifier cannot penetrate the stationary phase region, and they adsorb only on the outer surface [34]. In turn, the endcapping of the stationary phase decreases unwanted influence of accessible residual silanols on the separation process [33]. Thus, a better understanding of these phenomena could lead to the improvement of the prediction of separation selectivity changes with respect to the modifier choice.

The aim of the paper is to extend investigations on relative retention changes of aromatic hydrocarbons with polar groups in systems with hydro-organic eluent and 10 various stationary phases with different hydrocarbon lengths (from 3 to 18) and coverage density. Such research will allow us to conclude whether the proposed approach to explaining retention changes is applicable to systems with stationary phases other than C18 type. The approach could be very useful for the optimization of separation conditions of various substances, not only aromatic hydrocarbons with polar groups.

## 2. Results and Discussion

Retention data for a set of 34 compounds were obtained for systems with different nonpolar stationary phases (C3, C8, and C18 types) and organic modifiers (MeOH, ACN, and THF) in water mobile phases. The chosen concentration of the modifiers ensures similar retention of benzene in all systems. Moreover, the concentration of the organic modifier in the eluent did not exceed 50%. In order to compare the obtained results, the retention of substances was correlated as log k_1_ versus log k_2_, where k_1_ and k_2_ are solute retention factors in systems 1 and 2, respectively (see Figures S1–S10). On the resulting plots, one could see that the solutes formed two separate lines, the first one for substances with proton-donor and proton-acceptor properties (A_H_ > 0, B_H_ > 0, where A_H_-hydrogen bond acidity, B_H_-hydrogen bond basicity) and the second one for compounds capable of interacting as an electron-pair donor (B_H_ > 0). The obtained parameters of linear equations and values of correlation coefficient are gathered in Table 1.

### 2.1. Comparison of Substance Retention and Selectivity in ACN and THF Systems with Different Adsorbents

Selectivity differences between the systems with acetonitrile and tetrahydrofuran result from the different properties of these modifiers. The THF molecule is flatter and has greater volume and surface area than the ACN molecule. Tetrahydrofuran solvates and organizes the stationary phase area to a greater extent in comparison with ACN. It also exhibits greater proton-accepting properties than ACN (B_H_ THF = 0.55, B_H_ ACN = 0.31) [46] and a higher refractive index (n_THF_ = 1.405; n_ACN_ = 1.342); therefore, it is characterized by greater ability for dispersion interactions than acetonitrile. The dipole moment of ACN is 3.45D, while it is 1.75D for THF [47].

The correlation line for substances with proton-donor and proto-acceptor properties is above the line for solutes with an electron-donor group (Table 1) for all columns investigated, which indicates their higher retention in a system with THF in comparison with ACN. Compounds with proton-donor properties have a greater tendency to interact with tetrahydrofuran, which has high proton-accepting property [46]. On the other hand, substances with abilities for interactions as a proton acceptor and dipolar interactions have higher retention in comparison with others in systems with acetonitrile. Acetonitrile is characterized by weak contribution to interaction as a proton acceptor and possesses high dipolar properties [48].

As can be seen in Table 1, the values of slope of lines for substances with proton-donor and proton-acceptor properties are similar for all columns of C18 type and slightly higher for columns of type C8 (except column 300 SB 8). The lines for compounds capable of interacting as an electron-pair donor have slopes very similar to systems with all tested columns, except C3 columns.

**Figure 1:**
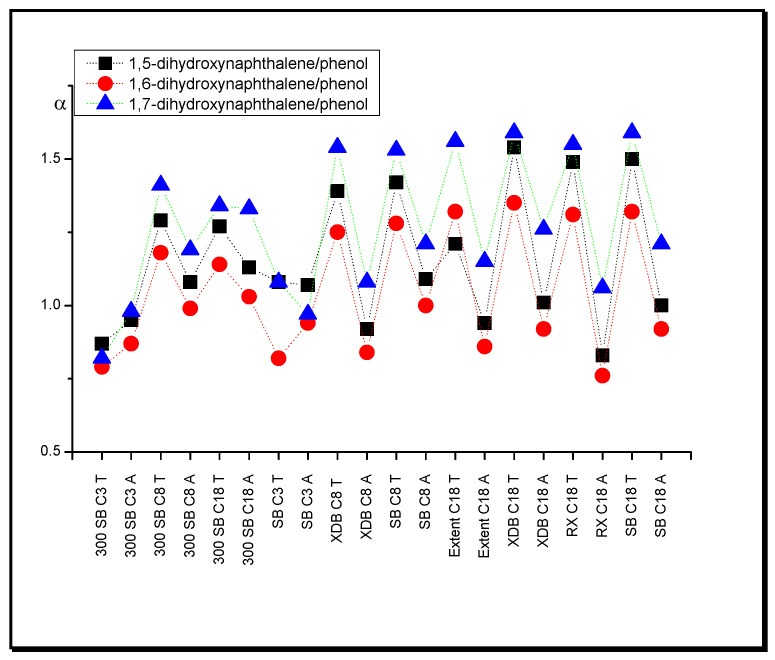
Separation factor, α, values of dihydroxynaphthalenes relative to phenol in systems with different nonpolar stationary phases and acetonitrile (A) in water and tetrahydrofuran (T) in water (modifier concentrations as in Table 1).

Based on retention data, one will notice that the substances with two OH groups (1,5-dihydroxynaphthalene, 1,6-dihydroxynaphthalene, 1,7-dihydroxynaphthalene) exhibit retention increase in comparison with the monofunctional solutes (phenol, *o*-cresol, *p*-cresol) in the THF system relative to that of ACN. This effect is caused by stronger hydrogen bond interaction of the solutes with tetrahydrofuran than with acetonitrile in the stationary phase region. The examples of separation factor (selectivity), α, values for naphthalene relative to phenol are presented in Figure 1.

It is worth underlining that this effect is not noticeable in systems with the SB C3 stationary phase and all 300 SB columns. In such cases, the separation factor of the compared compounds has almost identical values in the THF and ACN systems. As the length of the hydrocarbon chains decreases, less modifier takes part in their solvation, thus decreasing the intermolecular interactions of acetonitrile and tetrahydrofuran with the tested substances [49].

**Figure 2:**
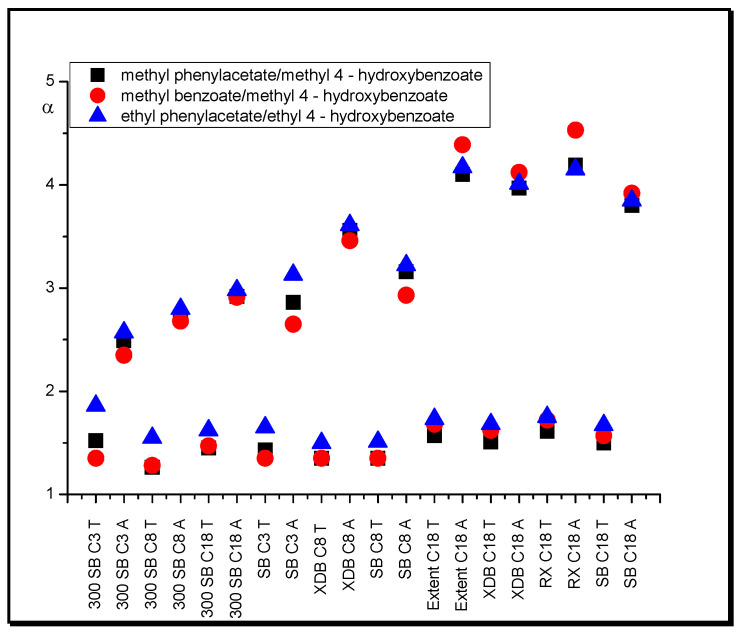
Separation factor, α, values of esters in systems with different nonpolar stationary phases and acetonitrile (A) in water and tetrahydrofuran (T) in water (modifier concentrations as in Table 1).

The influence of a hydroxyl functional group on substance retention can be observed when the retention of esters with a hydroxyl group (proton-donor group) is compared with the retention of those without this group. In systems with acetonitrile, the selectivity of the latter towards the former is quite high (from 2.5 to 4.2 for the pair methyl phenylacetate/methyl 4-hydroxybenzoate, from 1.9 to 4.5 for the pair methyl benzoate/methyl 4-hydroxybenzoate, from 2.6 to 4.2 for the pair ethyl phenylacetate/ethyl 4-hydroxybenzoate), and these values increase with increasing length of stationary phase chains (Figure 2). However, when tetrahydrofuran is used as a modifier, this value drops to approximately 1.5 for all columns. This proves the greater ability of THF to act as a proton acceptor in comparison with ACN [46].

Another example of selectivity changes demonstrates the 4-nitrobenzaldehyde/4-cyanobenzaldehyde pair. The higher value of the retention of the first substance in relation to the second one in systems with THF compared with those with ACN is due to the stronger interaction of the tetrahydrofuran molecule with the NO_2_ group. On the other hand, the CN group is characterized by strong dipolar properties and capability for dipolar interaction with ACN in the stationary phase region. For this reason, 4-cyanobenzaldehyde exhibits greater retention relative to 4-nitrobenzaldehyde in ACN systems. The values of separation factor are very close for systems with all stationary phases except 300 SB C3, 300 SB C8, and 300 SB C18.

The presence of a nitro group in the solute molecule enhances its retention relative to a substance without this group in THF relative to ACN systems. This phenomenon can be observed in such pairs of substances as 2-methylo-4-nitrophenol/*o*-cresol and 4-nitrophenol/phenol. It is worth noting that the ratio of separation coefficients for selected substance pairs in the THF system in relation to the ACN system has the highest values for systems with C18 columns except 300 SB C18 (1.4–1.7), intermediate values for SB C8 and XDB C8 (1.2–1.5), and smallest values for SB C3 and 300 SB C3, 300 SB C8, and 300 SB C18 (1.1–1.4). This means that as the length of the hydrocarbon chains increases, the retention of substances with an NO_2_ group in relation to a substance without this group increases in a system with THF compared with ACN. The nitro group possesses two highly polarized bonds with an electron density deficiency on the nitrogen atom. The THF molecule has two CO bonds with high electron density on the O atom. For this reason, relatively strong quadrupole interactions between nitrobenzene and THF take place [39].

**Figure 3:**
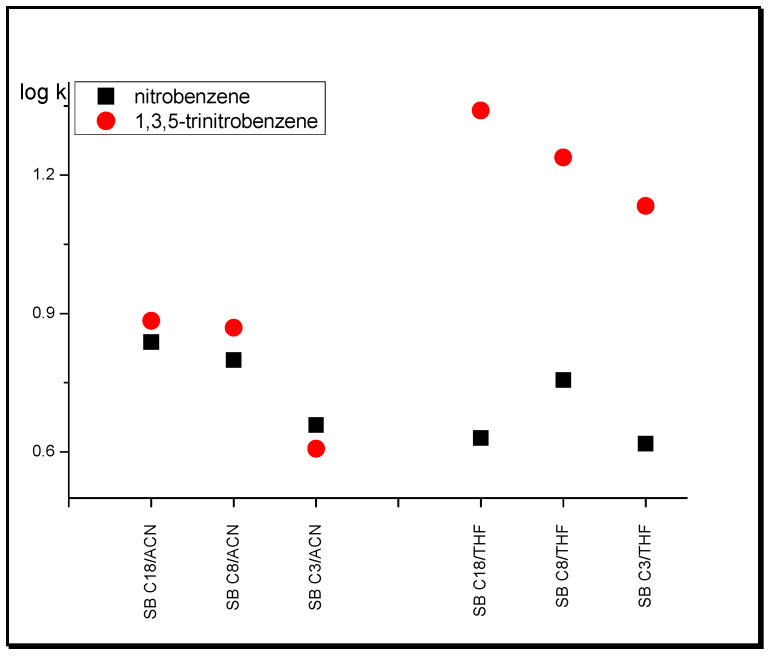
Log k of nitrobenzene and 1,3,5-trinitrobenzene in systems with different nonpolar stationary phases and tetrahydrofuran (THF) in water and acetonitrile (ACN) in water.

As the length of the hydrocarbon chains increases, more modifiers take part in their solvation, thus increasing the intermolecular interactions of acetonitrile and tetrahydrofuran with the tested substances [40]. These interactions are stronger the more NO_2_ groups the molecule has. Nitrobenzene derivatives with two nitro groups (i.e., 1,2-dinitrobenzene, 1,4-dinitrobenzene) exhibit higher retention than nitrobenzene but are much weaker than 1,3,5-trinitrobenzene in the THF system in comparison with ACN. The exemplification of this phenomenon is the retention of nitrobenzene and 1,3,5-trinitrobenzene in systems with SB C18, SB C8, and SB C3 stationary phases (Figure 3).

**Figure 4:**
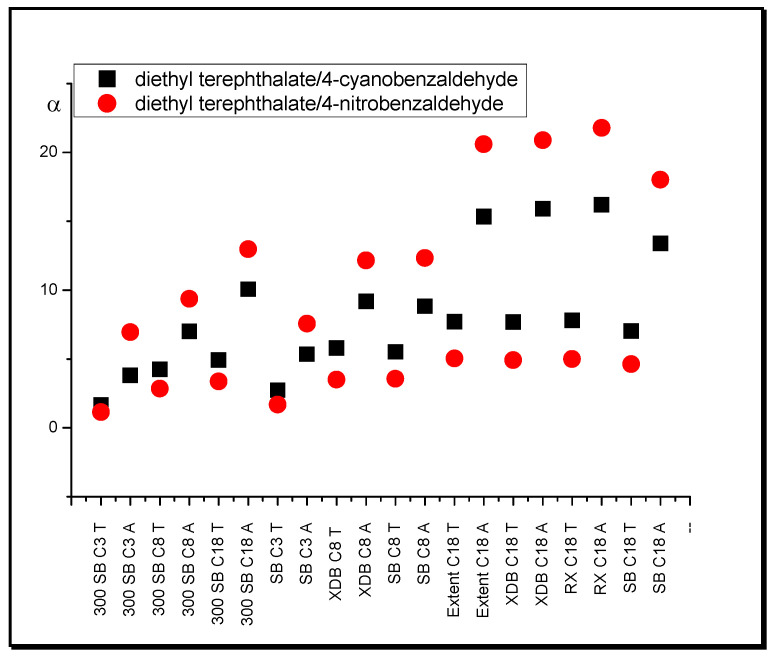
Separation factor, α, values of dimethyl isophthalate/4-cyanobenzaldehyde and dimethyl isophthalate/4-nitrobenzaldehyde in systems with different nonpolar stationary phases and (T) tetrahydrofuran in water, and (A) acetonitrile in water (modifier concentrations as in Table 1).

The influence of the shape of separated substance molecules on their retention and the selectivity of the separation, depending on the mobile phase modifier used, is also significant. In systems with tetrahydrofuran, compounds with a branched structure of molecules (isophthalates) show much lower retention relative to substances that possess a planar molecule in comparison with acetonitrile systems. This effect is visible for the following substance pairs: dimethyl isophthalate/4-cyanobenzaldehyde, dimethyl isophthalate/4-nitrobenzaldehyde, diethyl terephthalate/4-cyanobenzaldehyde, and diethyl terephthalate/4-nitrobenzaldehyde. In systems with acetonitrile, the values of the separation factor of these pairs of compounds are much higher relative to the systems with tetrahydrofuran. An increase of ordering of a surface region of the stationary phase is represented by the sequence of organic modifiers: MeOH, ACN, THF [49]. This means that relative retention of the solute with a more highly branched molecule structure decreases relative to the one with a less branched structure according to the order of the systems presented. In Figure 4, the relative selectivity changes for dimethyl isophthalate/4-cyanobenzaldehyde and dimethyl isophthalate/4-nitrobenzaldehyde are presented. One can see that differences between values of separation factor in THF and ACN systems rise with chain length and coverage density increase of the stationary phase.

### 2.2. Comparison of Substance Retention and Selectivity in MeOH and THF Systems with Different Adsorbents

Methanol is characterized by high proton-acceptor properties (B_H_ MeOH = 0.62) [46]. Methanol can also be a proton donor (A_H_ MeOH = 0.93). Moreover, the MeOH molecule is much smaller than the THF molecule and is less adsorbed on/in the stationary phase and therefore organizes the stationary phase region to a lesser extent than THF [49]. These characteristics are the reason for the differences in the relative retention of the tested substances in the discussed systems.

Similarly, as in the case of ACN–THF systems, the retention of substances has been correlated as log k_1_ (obtained in systems with THF) versus log k_2_ (obtained in systems with MeOH). It can be observed that, as in the ACN system versus the THF system, the solutes form two separate lines, one for substances with proton-donor and proton-acceptor properties and the other for compounds not capable of interacting as a proton donor (A_H_ = 0) (see Figures S11–S20). In the case of systems with the C18 adsorbent, the correlation line for substances with proton-donor groups is slightly above the line for compounds without these groups. The explanation for this fact is related to the greater sorption of THF in/on the stationary phase compared with that of MeOH.

This effect is also present when comparing systems (for all tested stationary phases) with tetrahydrofuran and acetonitrile, but the distances between the correlation lines are greater relative to the MeOH system versus the THF system.

Moreover, regarding the THF system versus the MeOH system, this difference is practically absent for C8 (80 Å) phases, or the line position is reversed for C3, C8, and C18 phases with a pore size of 300 Å. This phenomenon is probably due to solvent properties, which have proton-accepting properties and can form hydrogen bonds with the analytes [46]. The longer are the hydrocarbon chains of the stationary phases and the greater is their specific surface area (adsorbents with a larger pore diameter have a lower specific surface area), the more solvent can be adsorbed in/on its surface [40]. Shortening the length of the stationary phase ligands reduces the amount of THF in this region. The lower amount of tetrahydrofuran in this zone reduces the likelihood of hydrogen bond interactions with proton-donor substances. However, MeOH, contrary to ACN and THF, may be strongly adsorbed on the silica surface through hydrogen bonding with residual accessible silanols [40], apart from the solvation of hydrocarbon chains. Therefore, methanol sorption in the area of the stationary phase does not decrease as much as in case of THF with the shortening of the chain length. Moreover, the shorter are adsorbent hydrocarbon chains, the easier is the access of MeOH molecules to silanol groups [40].

**Figure 5:**
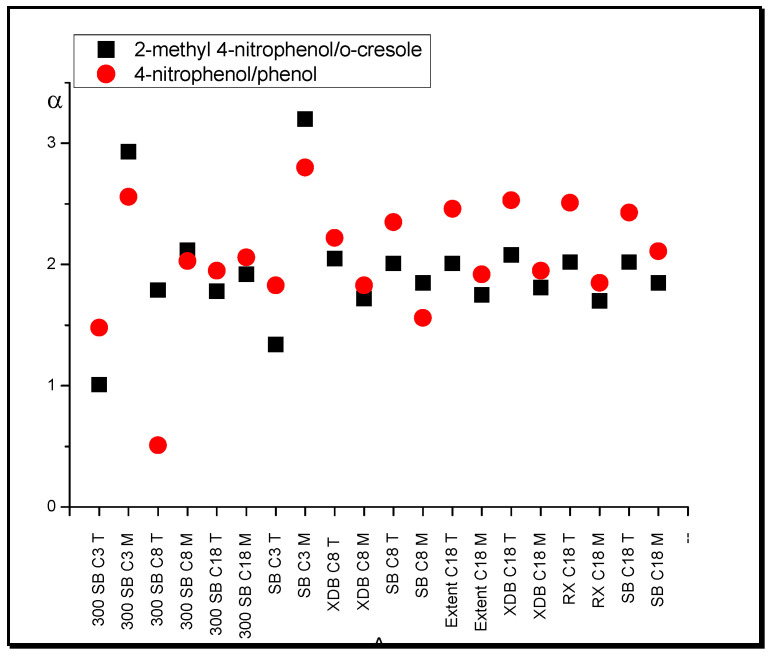
Separation factor, α, values of hydrocarbons with different functional groups in systems with different nonpolar stationary phases and methanol (M) in water and tetrahydrofuran (T) in water (modifier concentrations as in Table 1).

It can be observed that nitrophenols show a higher retention relative to phenols in systems with tetrahydrofuran compared with that with methanol. Examples of these relative retention changes for selected pairs of compounds are presented in Figure 5. This effect is visible for all C8 and C18 type columns.

When the C3 type or 300 SB C18 and 300 SB C8 stationary phases are used, the relationship is reversed and the values of the retention of nitrophenol relative to phenol are higher in the MeOH system compared with THF. This is probably due to the reduction in the amount of THF in the stationary phase area. The adsorbed amount of organic modifiers strongly depends on the stationary phase chain length and surface area. The shorter chain length (or smaller surface area) leads to less amount of THF in the stationary phase zone [40].

Methanol has the ability to solvate silanol groups, which are “more accessible” to those with shorter carbon chains [50]. Therefore, the concentration of MeOH in the area of the stationary phase does not decrease much, and therefore, it is possible to have a greater participation of intermolecular interactions of this modifier with nitrophenols relative to monophenols in a system with MeOH in comparison with THF.

Additionally, the influence of the number of -NO_2_ groups in a solute molecule on separation selectivity changes, depending on whether THF or MeOH was used as the mobile phase modifier, can be observed. In systems with methanol, the order of elution is consistent with the solvophobic theory [51]: as more polar groups (in this case -NO_2_) are present, a solute is less retained in the system with a hydrophobic stationary phase. In the system with THF, 1,3,5-trinitrobenzene has a much higher retention relative to 1,2-dinitrobenzene or nitrophenols. The reason for this are the electrostatic interactions of THF molecules with nitro groups (quadrupole interactions), as described above.

**Figure 6:**
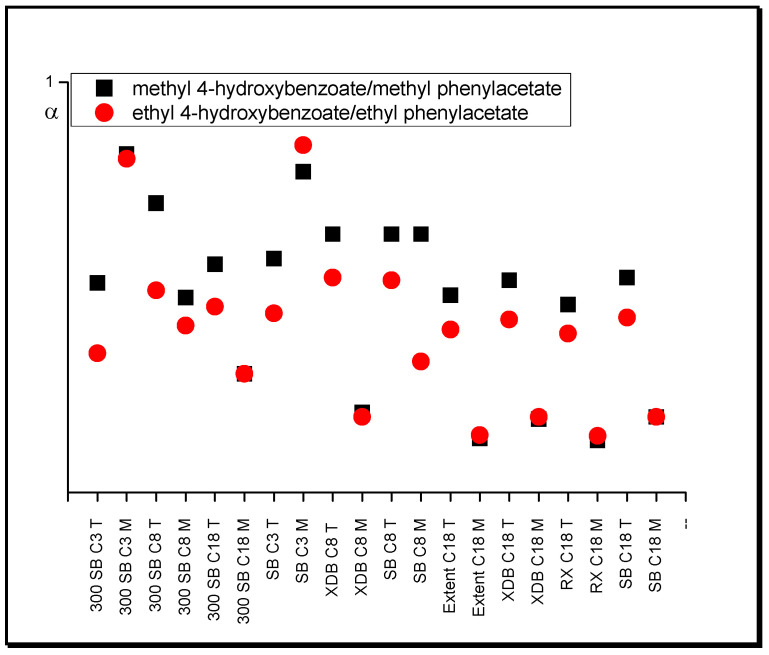
Separation factor, α, values of esters in systems with different nonpolar stationary phases and methanol (M) in water and tetrahydrofuran (T) in water (modifier concentrations as in Table 1).

Another example of changes in separation selectivity with the replacement of one modifier by another occurs when comparing the ester retention with and without the additional hydroxyl group. Esters that possess a hydroxyl group exhibit greater retention relative to esters without such group in systems with THF compared with systems with MeOH (Figure 6).

A similar effect, however, more pronounced, is observed for the correlation of THF versus ACN. This effect is present for systems with all tested stationary phases except C3, while for systems with all C8 and 300 SB C18 stationary phases, this effect is less visible. This phenomenon can be explained in a similar way as that for the nitrophenol/phenol selectivity changes discussed above.

**Figure 7:**
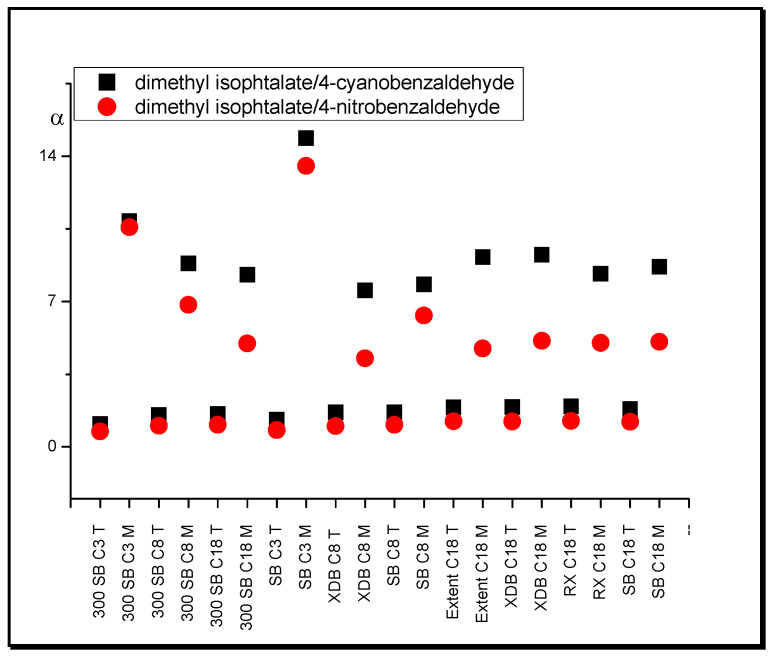
Separation factor, α, values of hydrocarbons with different functional groups in systems with different nonpolar stationary phases and methanol (M) in water and tetrahydrofuran (T) in water (modifier concentrations as in Table 1).

Another interesting effect of separation selectivity changes on the mobile phase modifier is observed for the substance pairs dimethyl isophthalate/4-cyanobenzaldehyde and dimethyl isophthalate/4-nitrobenzaldehyde. As can be seen in Figure 7, separation factor values are much higher in the systems with MeOH relative to THF. A similar effect was also observed for THF versus ACN systems, but the differences were much smaller.

It is worth underlining that the ability of a modifier to increase the ordering of the hydrocarbon stationary phase rises in the following order: MeOH, ACN, THF [49]. For this reason, in the system with MeOH, the stationary phase is more accessible for entropic penetration by branched molecules relative to the system with THF.

A stationary phase in the system with THF is more ordered than in that with ACN and, especially, the one containing MeOH [49]. Molecules with a branched structure (e.g., dimethyl isophthalate) exhibit lower entropic penetration of this stationary phase region relative to molecules of planar structure (4-cyanobenzaldehyde, 4-nitrobenzaldehyde).

### 2.3. Comparison of Substance Retention and Selectivity in MeOH and ACN Systems with Different Adsorbents

Adsorption of acetonitrile molecules on chemically bonded phases is caused by their interaction with bonded ligands and dipole–dipole interactions, while their interactions with residual silanols by hydrogen bonding are weak. Methanol may be adsorbed near the silica surface by hydrogen bonding and dipole–dipole interaction with residual accessible silanols [40]. Therefore, the low-coverage-density phase is better solvated by methanol molecules than by acetonitrile [52]. The C18 stationary phase in the system with ACN is more ordered than with MeOH [53]. However, changes in separation selectivity between these modifier systems regarding the shape of the molecules are much smaller than between the systems of these modifiers relative to THF.

The solutes, as in the ACN system versus the THF system, form two separate lines, one for substances with groups of proton-donor and electron-pair-donor (proton-acceptor) characters and the second for compounds with groups of electron-pair donors. However, contrary to the compared ACN and THF systems, the correlation line for analytes that can act only as a proton acceptor are placed above the first line, which means that these compounds show higher retention in relation to other substances in systems with acetonitrile (see Figures S21–S30). This is due to the stronger intermolecular interaction of ACN with these substances in the stationary phase region compared with MeOH [46]. On the other hand, substances that possess groups with both proton-donor and proton-acceptor properties show relatively stronger interactions with methanol.

The influence of the number of –NO_2_ groups on separation selectivity changes, depending on the organic modifier used, can be observed. In systems with methanol, especially in the case of C18 and C8 columns, the order of elution is consistent with the solvophobic theory [50]: the larger the number of -NO_2_ groups a molecule has, the less it is retained. However, in a system with ACN, all nitro derivatives exhibit similar retention. This phenomenon is probably caused by stronger dipole–dipole interactions of the discussed compounds with ACN relative to the MeOH system.

**Figure 8:**
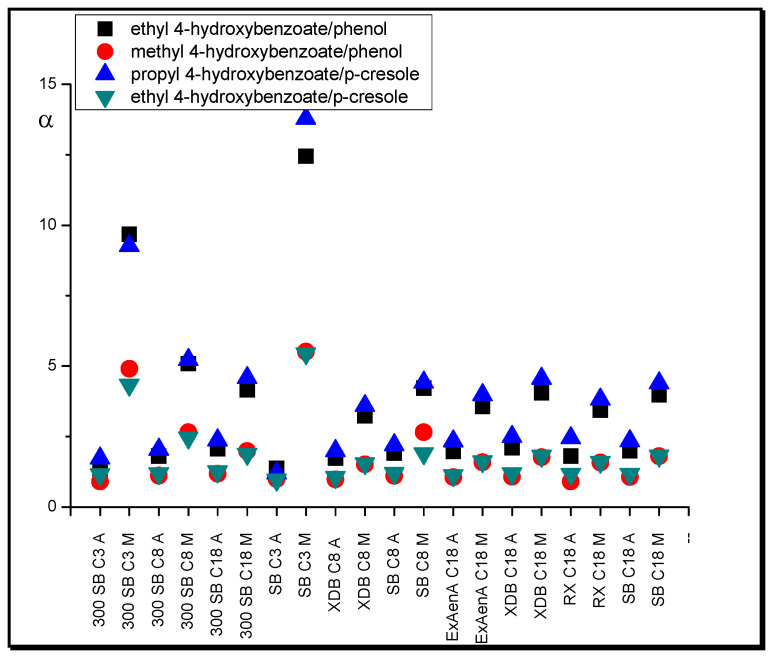
Separation factor, α, values of hydrocarbons with different functional groups in systems with different nonpolar stationary phases and methanol (M) in water and acetonitrile (A) in water (modifier concentrations as in Table 1).

An increase in the relative retention of phenols with an ester group in relation to phenols in systems with methanol compared with systems with acetonitrile stands as another example of changes in separation selectivity in respect of the shape of the molecules (Figure 8). Phenols with an ester group (methyl, ethyl, and propyl 4-hydroxybenzoate) have a more branched molecular shape than monophenols (phenol, *o*-cresol), and their entropic penetration of the stationary phase region in a system with ACN is more restricted than with MeOH. Methanol orders organic ligands to a lesser extent; therefore, in systems with this modifier, molecules with branched shapes have a greater possibility of entropic penetration, and their relative retention is higher [49].

An interesting effect can be seen when comparing the correlation plots for ACN versus MeOH and THF versus MeOH. There is a decrease in the slope of the correlation lines with the shortening of the hydrocarbon chain length and with an increase in the pore diameter.

This phenomenon is especially visible for the systems with columns of SB type. For the THF system versus MeOH system, the slope of the correlation lines is smaller than for ACN versus MeOH, and for both, it is much less than one.

This reduction in the slope means that the range of retention changes of the tested substances in the THF and ACN systems is smaller compared with the MeOH system. It is probably caused by the reduction of intermolecular interactions of acetonitrile and tetrahydrofuran with the substances in the stationary phase region (the shorter hydrocarbon chains, then the less modifier in the stationary phase region) [40]. In the case of systems with methanol, the reduction of its content in the area of the stationary phase is not essential because, as already mentioned, apart from solvation of carbon chains, it can also form hydrogen bonds with residual silanol groups [34].

## 3. Materials and Methods

Solutes (Table 2, Table S1) were obtained from different sources. Benzene (≥99.9%), toluene (≥99.8%), phenol (≥99%), *o*-cresol (≥99%), *p*-cresol (≥99%), 2-naphthol (≥99%), acetophenone (≥99%), nitrobenzene (≥99%), 4-cyanobenzaldehyde (≥98%), benzonitrile (≥99%), 1,5-dihydroxynaphthalene (≥97%), 1,6-dihydroxynaphthalene (≥99%), 2-cyanophenol (≥99%), dimethyl-4,4′-diphenyl dicarboxylate (≥99%), ethyl 4-hydroxybenzoate (≥99%), methyl 4-hydroxybenzoate (≥99%), propyl 4-hydroxybenzoate (≥99%), dimethyl isophthalate (≥99%), 4-nitrobenzaldehyde (≥98%), 4-nitrobenzyl alcohol (≥99%), 2-nitrophenol (≥98%), 3-nitrophenol (≥99%), 2-nitro-4-chlorophenol (≥97%), 2-methylo-4-nitrophenol (≥97%), 4-nitrophenol (≥99%), 1-chloro-2,4-dinitrobenzene (≥97%), 1,2-dinitrobenzene (≥99%), 1,4-dinitrobenzene (≥98%), methyl phenylacetate (≥98%), ethyl phenylacetate (≥98%), and 1,3,5-trinitrobenzene (analytical standard) were supplied by Sigma-Aldrich (St. Louis, MO, USA). 1,7-Dihydroxynaphthalene (≥97%) and diethyl terephthalate (≥95%) were supplied by Alfa Aesar (Heysham, Lancashire, UK), and methyl benzoate (analytical standard) was supplied by Fluka (Buchs, Switzerland). All solvents (methanol, acetonitrile, and tetrahydrofuran), HPLC grade, were purchased from Merck (Darmstadt, Germany). Water was bidistilled. Eluents contained 0.1% acetic acid (analytical grade, Merck).

Measurements of retention were performed with the 1290 Agilent Infinity LC System (Santa Clara, CA, USA) equipped with a DAD detector. The C3, C8, and C18 columns were used in the study. The properties of the stationary phases are listed in Table 3. The columns were thermostated at 20 °C. The flow rate was set at 0.5 mL/min, the injection volume was 10 μL, and the eluate was monitored at 254 nm. All experiments were performed in triplicate.

## 4. Conclusions

Retention correlation and comparison of aromatic hydrocarbons with a polar group in methanol, acetonitrile, and tetrahydrofuran systems demonstrate differences in separation selectivity between these systems. Moreover these differences are closely related to the stationary phase used. The results presented in the paper confirm our previous presumption that the explanation of separation selectivity changes in reversed-phase systems, when one modifier is replaced by another in the mobile phase, can be performed, taking into account molecular interactions of the solutes with the stationary phase components, especially mobile phase modifier (organic solvent) extracted into the stationary phase. Moreover, these results point out that the previously described approach can also be applied to systems with other nonpolar adsorbents of the C3 and C8 types, keeping in mind that the adsorbed amount of organic modifiers strongly depends on the chain length of the stationary phase. Additionally, one has to take into account that the organic modifiers interact with free silanol groups at different extents.

## Figures and Tables

**Table 1:** The obtained parameters of linear equations and values of correlation coefficient for investigated chromatographic systems.

	**SB C18**			**Rx C18**			**XDB C18**			**Extend C18**	
	BH > 0	AH > 0, BH > 0		BH > 0	AH > 0, BH > 0		BH > 0	AH > 0, BH > 0		BH > 0	AH > 0, BH > 0
35% ACN (k_1_) 44% MeOH (k_2_)	a = 0.6811	a = 0.6626	34% ACN (k_1_)	a = 0.7504	a = 0.7114	34% ACN (k_1_)	a = 0.7749	a = 0.6837	35% ACN (k_1_)	a = 0.7819	a = 0.6867
	b = 0.3047	b = 0.0626	-	b = 0.1614	b = 0.0307	-	b = 0.1871	b = 0.0397	-	b = 0.1734	b = 0.0534
	R = 0.7946	R = 0.9541	45% MeOH (k_2_)	R = 0.9	R = 0.9630	43% MeOH (k_2_)	R = 0.9048	R = 0.9575	32% MeOH (k_2_)	R = 0.9239	R = 0.9633
32% THF (k_1_) 35% ACN (k_2_)	a = 0.5507	a = 0.7765	32% THF (k_1_)	a = 0.5938	a = 0.7513	31%THF (k_1_)	a = 0.6729	a = 0.7607	31% THF (k_1_)	a = 0.6447	a = 0.7578
	b = 0.3885	b = 0.4242	-	b = 0.2975	b = 0.4005	-	b = 0.2640	b = 0.4519	-	b = 0.3460	b = 0.4494
	R = 0.5846	R = 0.8414	34% ACN (k_2_)	R = 0.7220	R = 0.8296	34% ACN (k_2_)	R=0.7876	R = 0.8413	35% ACN (k_2_)	R = 0.6876	R = 0.8321
32% THF (k_1_) 44% MeOH (k_2_)	a = 0.491	a = 0.4765	32% THF (k_1_)	a= 0.4060	a = 0.5040	31% THF (k_1_)	a = 0.5007	a = 0.4783	31% THF (k_1_)	a = 0.5239	a = 0.4904
	b = 0.4602	b = 0.4964	-	b = 0.4252	b = 0.4375	-	b = 0.4063	b = 0.5099	-	b = 0.44	b = 0.5077
	R = 0.6080	R = 0.7434	45% MeOH (k_2_)	R = 0.5921	R = 0.7534	45%MeOH (k_2_)	R = 0.6879	R = 0.7407	45% MeOH (k_2_)	R = 0.6603	R = 0.7554
	**SB C8**			**XDB C8**			**SB C3**				
	BH > 0	AH > 0, BH > 0		BH > 0	AH > 0, BH > 0		BH > 0	AH > 0, BH > 0			
35% ACN (k_1_) 42% MeOH (k_2_)	a = 0.5695	a = 0.59	36% ACN (k_1_)	a = 0.6426	a = 0.6109	33% ACN (k_1_)	a = 0.6249	a = 0.2294			
	b = 0.225	b = 0.0002	-	b = 0.2653	b = 0.0606	-	b = 0.0761	b = 0.1295			
	R = 0.7892	R = 0.8612	45% MeOH (k_2_)	R = 0.834	R = 0.9557	25% MeOH (k_2_)	R = 0.6987	R = 0.4205			
33% THF (k_1_) 35% ACN (k_2_)	a = 0.6870	a = 0.8771	32% THF (k_1_)	a = 0.6699	a = 0.9109	33% THF (k_1_)	a = 0.1515	a = 0.613			
	b = 0.289	b = 0.3277	-	b = 0.3413	b = 0.3867	-	b = 0.531	b = 0.439			
	R = 0.6504	R = 0.8371	36% ACN	R = 0.658	R = 0.7837	33% ACN (k_2_)	R = 0.2593	R = 0.387			
33% THF (k_1_) 42% MeOH (k_2_)	a = 0.3191	a = 0.4338	32% THF (k_1_)	a = 0.4129	a = 0.5325	33% THF (k_1_)	a = 0.1293	a = 0.2026			
	b = 0.5135	b = 0.3964	-	b = 0.5321	b = 0.4558	-	b = 0.4538	b = 0.4203			
	R = 0.4187	R = 0.6044	45% MeOH (k_2_)	R = 0.5264	R = 0.7164	25% MeOH (k_2_)	R = 0.2571	R = 0.3876			

**Table 2:** List of investigated solutes and their descriptors [54].

Substance	A_H_, Hydrogen Bond Acidity *	B_H_, Hydrogen Bond Basicity *	Substance	A_H_, Hydrogen Bond Acidity *	B_H_, Hydrogen Bond Basicity *
1. Benzene	0	0.14	18. 3-Nitrophenol	0.79	0.23
2. Toluene	0	0.14	19. 4-Nitrophenol	0.82	0.26
3. Phenol	0.6	0.3	20. 2-Methyl-4-nitrophenol	0.78	0.25
4. 2-Cresol	0	0.14	21. Methyl 4-hydroxybenzoate	0.69	0.45
5. 4-Cresol	0.52	0.3	22. Ethyl 4-hydroxybenzoate	0.69	0.45
6. 2-Naphthol	0.57	0.31	23. Propyl 4-hydroxybenzoate	0.69	0.45
7. Methyl phenylacetate	0.61	0.4	24. 4-Nitrobenzyl alcohol	0.44	0.62
8. Ethyl phenylacetate	0	0.58	25. 1,2-Dinitrobenzene	0	0.38
9. Methyl benzoate	0	0.57	26. 1,4-Dinitrobenzene	0	0.46
10. Acetophenone	0	0.46	27. 1-Chloro-2,4-dinitrobenzene	0	0.42
11. Nitrobenzene	0	0.28	28. 4-Nitrobenzaldehyde	0	0.44
12. Benzonitrile	0	0.33	29. 4-Cyanobenzaldehyde	0	0.33
13. 1,5-Dihydroxynaphthalene	0.93	0.58	30. Dimethyl isophthalate	0	0.67
14. 1,6-Dihydroxynaphthalene	0.98	0.57	31. Diethyl terephthalate		
15. 1,7-Dihydroxynaphthalene	1.02	0.54	32. Dimethyl 4,4-diphenylcarboxylate	0	0.91
16. 2-Cyanophenol	0.78	0.34	33. 2-Nitro-4-chlorophenol	0.1	0.3
17. 2-Nitrophenol	0.05	0.37	34. 1,3,5-Trinitrobenzene	0	0.6

* Experimentally determined, from various sources.

**Table 3:** Characterization of used columns.

No.	Column	Dimensions (mm)	Particle Diameter (µm)	Pore Diameter (Å)	Surface Area (m^2^/g)	Endcapped	Carbon Load (%)	Coverage Density (µmol/m^2^)
1	Zorbax SB C18	3 × 150	5	80	180	no	10	2.98
2	Zorbax Rx C18	3 × 150	5	80	180	no	12	2.98
3	Zorbax Eclipse XDB C18	3 × 150	5	80	180	yes	10	4
4	Zorbax Extend C18	3 × 150	5	80	180	yes	12.5	3.8
5	Zorbax SB C8	3 × 150	5	80	180	no	5.5	2.4
6	Zorbax Eclipse XDB C8	3 × 150	5	80	180	yes	7.6	3.8
7	ProntoSIL C4	3 × 125	5	120	300	yes	5	-
8	Zorbax SB C3	3 × 150	5	80	180	no	4	-
9	Zorbax 300SB C18	3 × 150	5	300	45	no	2.8	-
10	Zorbax 300SB C8	3 × 150	5	300	45	no	1.5	-
11	Zorbax 300SB C3	3 × 150	5	300	45	no	1.1	-

## Supplementary Materials

The following are available online, Figure S1. Log k in 32% *v*/*v* THF plotted against log k in 35% *v*/*v* ACN. SB C18 stationary phase. Solute numbers as in Table 2. Figure S2. Log k in 32% *v*/*v* THF plotted against log k in 34% *v*/*v* ACN. Rx C18 stationary phase. Solute numbers as in Table 2. Figure S3. Log k in 31% *v*/*v* THF plotted against log k in 34% *v*/*v* ACN. Eclipse XDB C18 stationary phase. Solute numbers as in Table 2. Figure S4. Log k in 31% *v*/*v* THF plotted against log k in 35% *v*/*v* ACN. Extend C18 stationary phase. Solute numbers as in Table 2. Figure S5. Log k in 33% *v*/*v* THF plotted against log k in 35% *v*/*v* ACN. SB C8 stationary phase. Solute numbers as in Table 2. Figure S6. Log k in 32% *v*/*v* THF plotted against log k in 36% *v*/*v* ACN. Eclipse XDB C8 stationary phase. Solute numbers as in Table 2. Figure S7. Log k in 33% *v*/*v* THF plotted against log k in 33% *v*/*v* ACN. SB C3 stationary phase. Solute numbers as in Table 2. Figure S8. Log k in 35% *v*/*v* THF plotted against log k in 34% *v*/*v* ACN. 300 SB C18 stationary phase. Solute numbers as in Table 2. Figure S9. Log k in 35% *v*/*v* THF plotted against log k in 36% *v*/*v* ACN. 300 SB C8 stationary phase. Solute numbers as in Table 2. Figure S10. Log k in 34% *v*/*v* THF plotted against log k in 32% *v*/*v* ACN. 300 SB C3 stationary phase. Solute numbers as in Table 2. Figure S11. Log k in 32% *v*/*v* THF plotted against log k in 44% *v*/*v* MeOH. SB C18 stationary phase. Solute numbers as in Table 2. Figure S12. Log k in 32% *v*/*v* THF plotted against log k in 45% *v*/*v* MeOH. Rx C18 stationary phase. Solute numbers as in Table 2. Figure S13. Log k in 31% *v*/*v* THF plotted against log k in 43% *v*/*v* MeOH. Eclipse XDB C18 stationary phase. Solute numbers as in Table 2. Figure S14. Log k in 31% *v*/*v* THF plotted against log k in 45% *v*/*v* MeOH. Extend C18 stationary phase. Solute numbers as in Table 2. Figure S15. Log k in 33% *v*/*v* THF plotted against log k in 42% *v*/*v* MeOH. SB C8 stationary phase. Solute numbers as in Table 2. Figure S16. Log k in 32% *v*/*v* THF plotted against log k in 45% *v*/*v* MeOH. XDB C8 stationary phase. Solute numbers as in Table 2. Figure S17. Log k in 33% *v*/*v* THF plotted against log k in 25% *v*/*v* MeOH. SB C3 stationary phase. Solute numbers as in Table 2. Figure S18. Log k in 35% *v*/*v* THF plotted against log k in 44% *v*/*v* MeOH. 300 SB C18 stationary phase. Solute numbers as in Table 2. Figure S19. Log k in 35% *v*/*v* THF plotted against log k in 41% *v*/*v* MeOH. 300 SB C8 stationary phase. Solute numbers as in Table 2. Figure S20. Log k in 33% *v*/*v* ACN plotted against log k in 26% *v*/*v* MeOH. 300 SB C3 stationary phase. Solute numbers as in Table 2. Figure S21. Log k in 35% *v*/*v* ACN plotted against log k in 44% *v*/*v* MeOH. SB C18 stationary phase. Solute numbers as in Table 2. Figure S22. Log k in 34% *v*/*v* ACN plotted against log k in 45% *v*/*v* MeOH. Rx C18 stationary phase. Solute numbers as in Table 2. Figure S23. Log k in 34% *v*/*v* ACN plotted against log k in 43% *v*/*v* MeOH. Eclipse XDB C18 stationary phase. Solute numbers as in Table 2. Figure S24. Log k in 35% *v*/*v* ACN plotted against log k in 45% *v*/*v* MeOH. Extend C18 stationary phase. Solute numbers as in Table 2. Figure S25. Log k in 35% *v*/*v* ACN plotted against log k in 42% *v*/*v* MeOH. SB C8 stationary phase. Solute numbers as in Table 2. Figure S26. Log k in 36% *v*/*v* ACN plotted against log k in 45% *v*/*v* MeOH. Eclipse XDB C8 stationary phase. Solute numbers as in Table 2. Figure S27. Log k in 33% *v*/*v* ACN plotted against log k in 25% *v*/*v* MeOH. SB C3 stationary phase. Solute numbers as in Table 2. Figure S28. Log k in 34% *v*/*v* ACN plotted against log k in 44% *v*/*v* MeOH. 300 SB C18 stationary phase. Solute numbers as in Table 2. Figure S29. Log k in 36% *v*/*v* ACN plotted against log k in 41% *v*/*v* MeOH. 300 SB C8 stationary phase. Solute numbers as in Table 2. Figure S30. Log k in 32% *v*/*v* ACN plotted against log k in 26% *v*/*v* MeOH. 300 SB C3 stationary phase. Solute numbers as in Table 2. Table S1. Structural formulas of substances used in experiments.
