# Supplementary material for: Comparison of the Retention and Separation Selectivity of Aromatic Hydrocarbons with Polar Groups in RP-HPLC Systems with Different Stationary Phases and Eluents
Source: Molecules. 2020 Nov 1;25(21):5070. doi: 10.3390/molecules25215070 (PMC7663032; doi:10.3390/molecules25215070)
Supplement: Supplementary file 1 [file molecules-25-05070-s001.pdf]

Article

Comparison of the Retention and separation Selectivity of Aromatic Hydrocarbons with Polar Groups in RP-HPLC Systems with Different Stationary Phases and Eluents

Anna Klimek-Turek [1,*], Beata Misiołek [1,2] and Tadeusz H. Dzido [1]

[1] Department of Physical Chemistry, Medical University of Lublin, Chodźki 4a, 20-093 Lublin, Poland; beata.misiolek@umlub.pl (B.M.); tadeusz.dzido@umlub.pl (T.H.D.)

[2] Department for Variations and Renewals of Medicinal Products, The Office for Registration of Medicinal Products, Medical Devices and Biocidal Products, Al. Jerozolimskie 181C, 02-222 Warsaw, Poland

* Correspondence: anna.klimek@umlub.pl, Tel.:+48 81448 7206

Academic Editors: Magdalena Wojciak-Kosior and Bogusław Buszewski

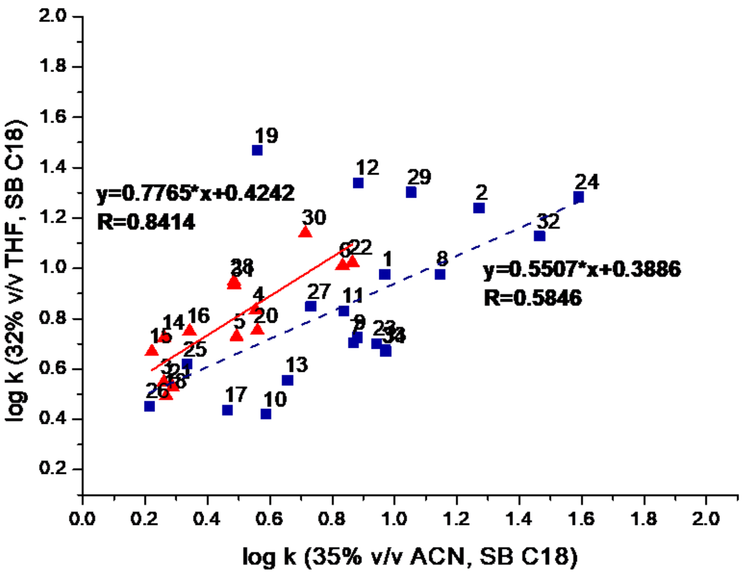

Figure S1. Log k in 32% v/v THF plotted against log k in 35% v/v ACN. SB C18 stationary phase. Solute numbers as in Table 2.

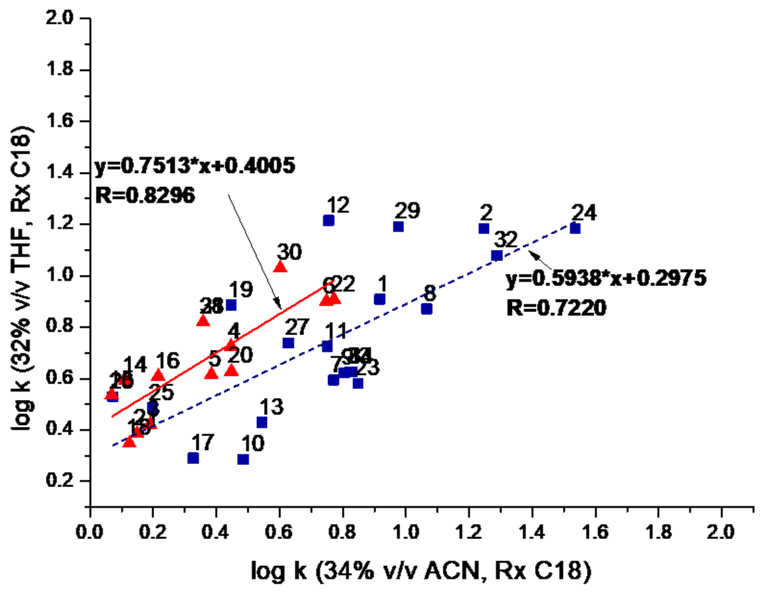

Figure S2. Log k in 32% v/v THF plotted against log k in 34% v/v ACN. Rx C18 stationary phase. Solute numbers as in Table 2.

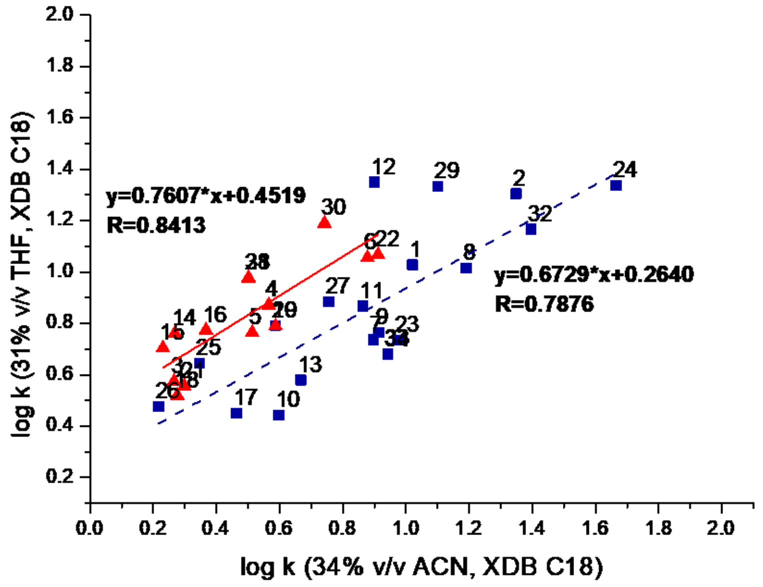

Figure S3. Log k in 31% v/v THF plotted against log k in 34% v/v ACN. Eclipse XDB C18 stationary phase. Solute numbers as in Table 2.

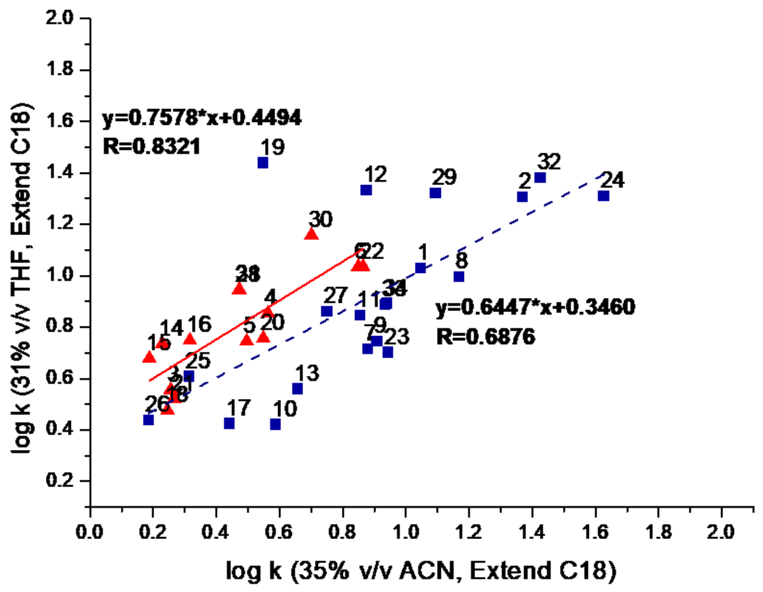

Figure S4. Log k in 31% v/v THF plotted against log k in 35% v/v ACN. Extend C18 stationary phase. Solute numbers as in Table 2.

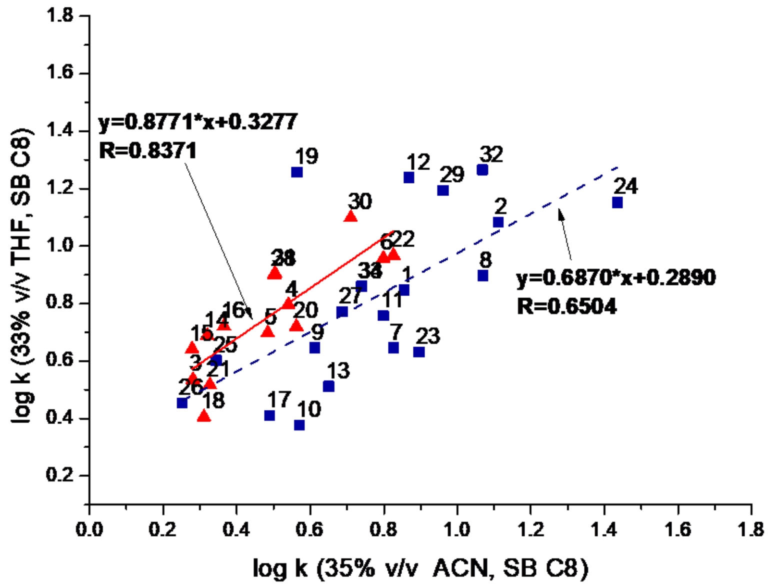

Figure S5. Log k in 33% v/v THF plotted against log k in 35% v/v ACN. SB C8 stationary phase. Solute numbers as in Table 2.

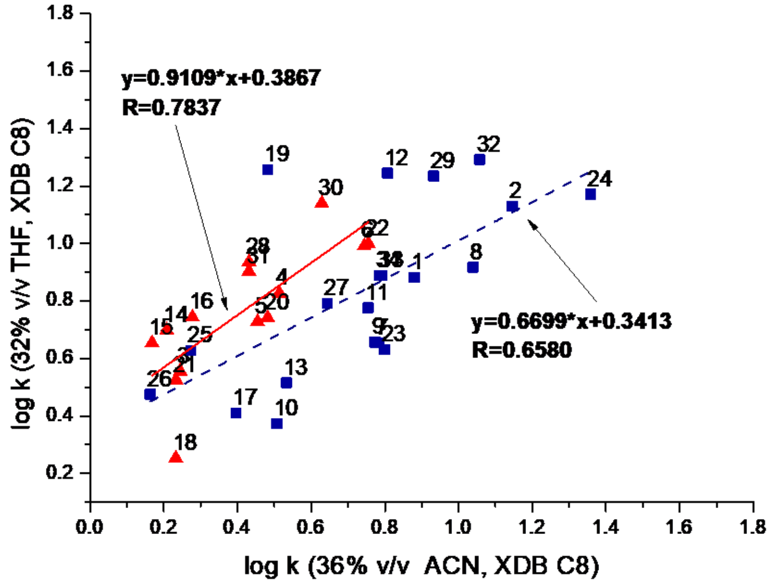

Figure S6. Log k in 32% v/v THF plotted against log k in 36% v/v ACN. Eclipse XDB C8 stationary phase. Solute numbers as in Table 2.

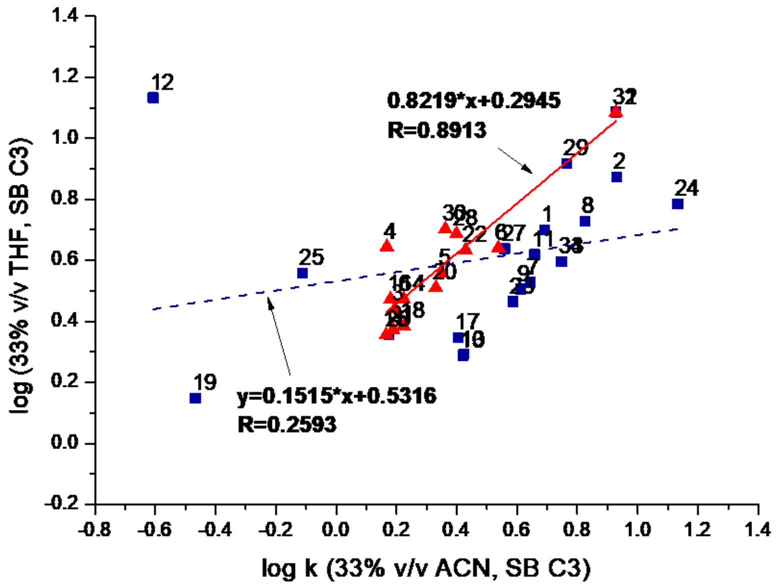

Figure S7. Log k in 33% v/v THF plotted against log k in 33% v/v ACN. SB C3 stationary phase. Solute numbers as in Table 2.

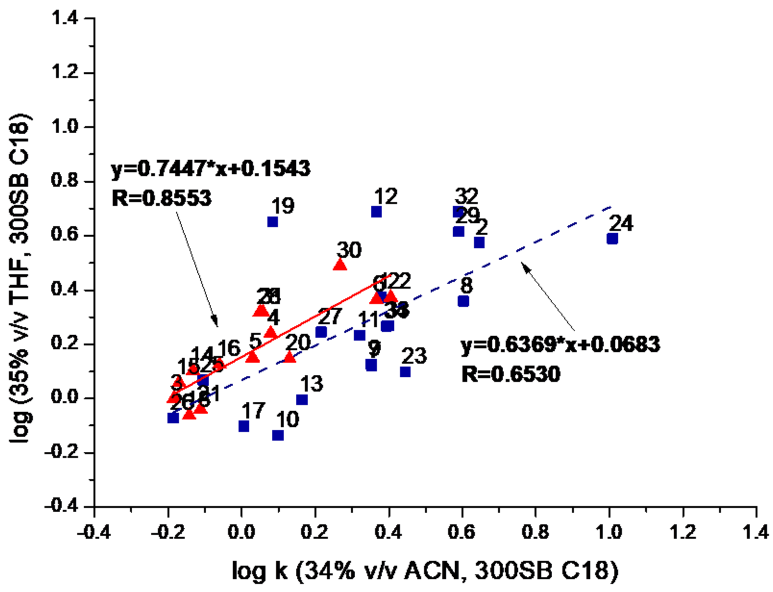

Figure S8. Log k in 35% v/v THF plotted against log k in 34% v/v ACN. 300 SB C18 stationary phase. Solute numbers as in Table 2.

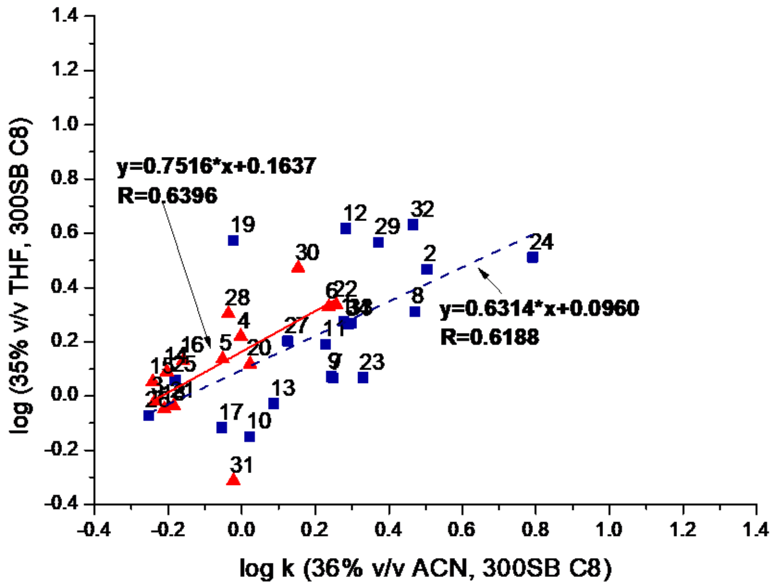

Figure S9. Log k in 35% v/v THF plotted against log k in 36% v/v ACN. 300 SB C8 stationary phase. Solute numbers as in Table 2.

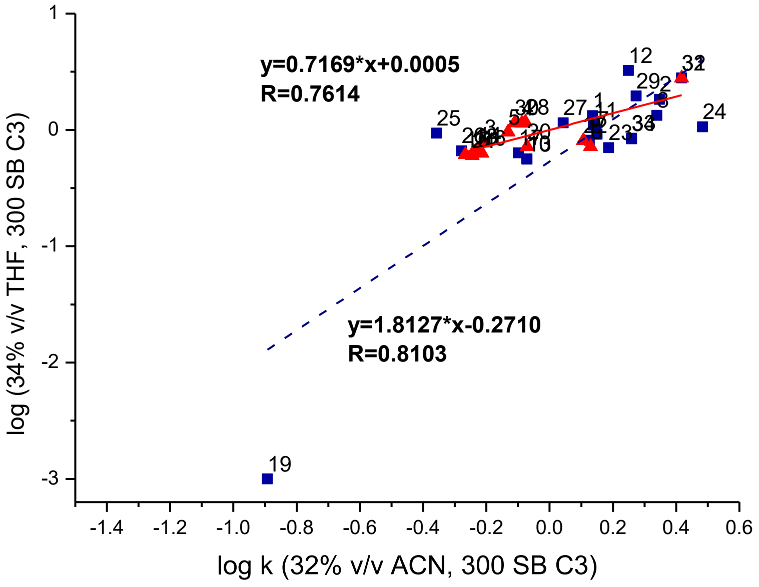

Figure S10. Log k in 34% v/v THF plotted against log k in 32% v/v ACN. 300 SB C3 stationary phase. Solute numbers as in Table 2.

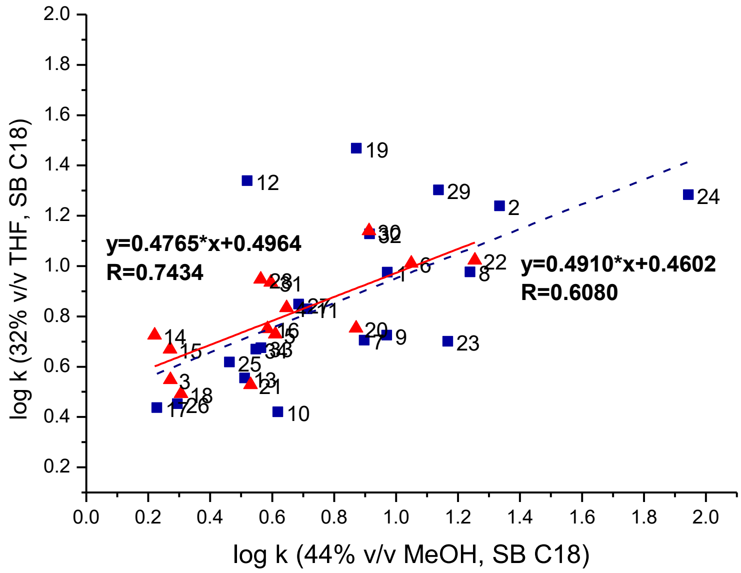

Figure S11. Log k in 32% v/v THF plotted against log k in 44% v/v MeOH. SB C18 stationary phase. Solute numbers as in Table 2.

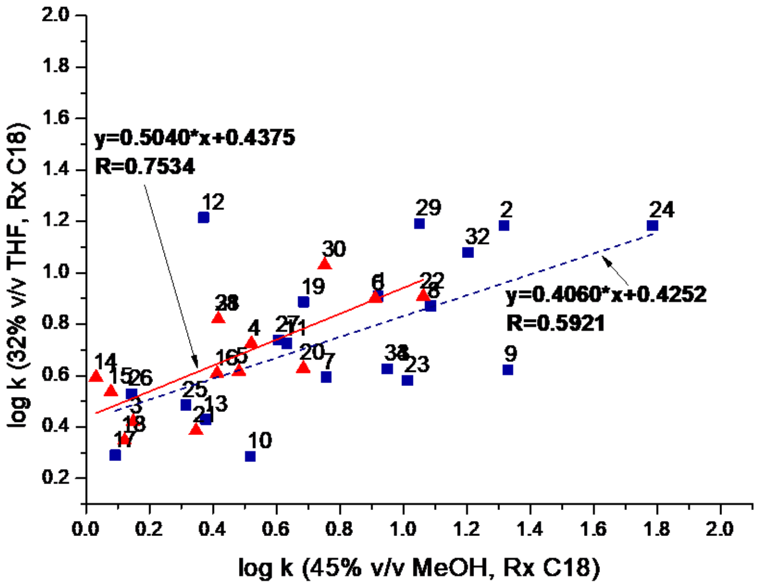

Figure S12. Log k in 32% v/v THF plotted against log k in 45% v/v MeOH. Rx C18 stationary phase. Solute numbers as in Table 2.

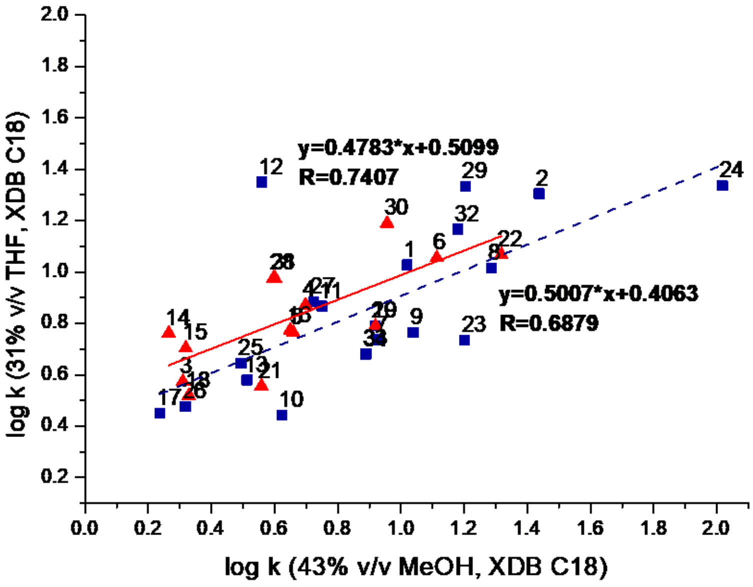

Figure S13. Log k in 31% v/v THF plotted against log k in 43% v/v MeOH. Eclipse XDB C18 stationary phase. Solute numbers as in Table 2.

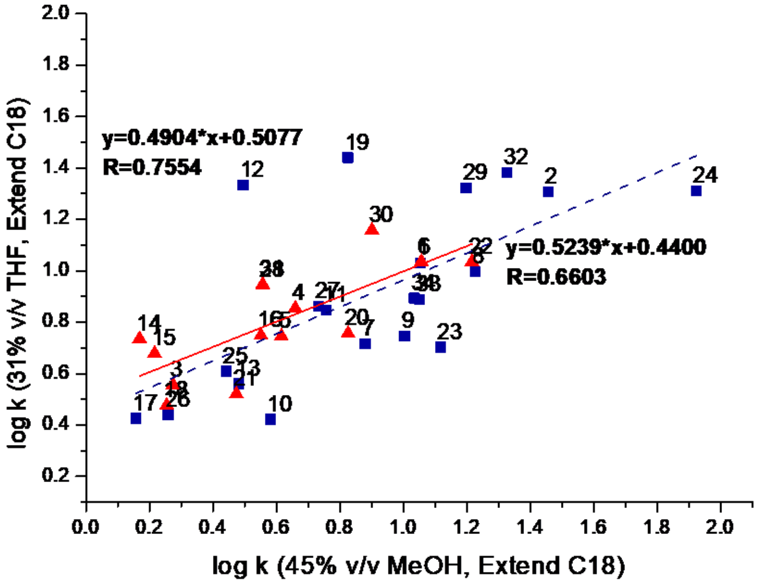

Figure S14. Log k in 31% v/v THF plotted against log k in 45% v/v MeOH. Extend C18 stationary phase. Solute numbers as in Table 2.

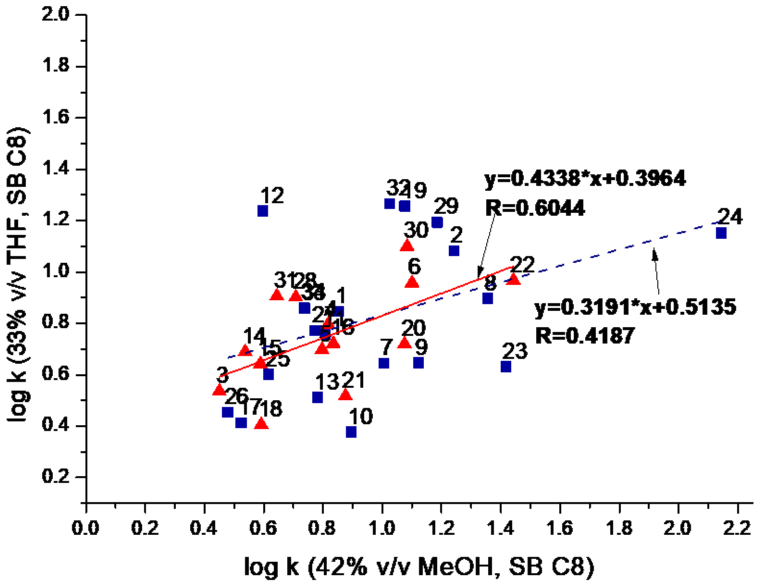

Figure S15. Log k in 33% v/v THF plotted against log k in 42% v/v MeOH. SB C8 stationary phase. Solute numbers as in Table 2.

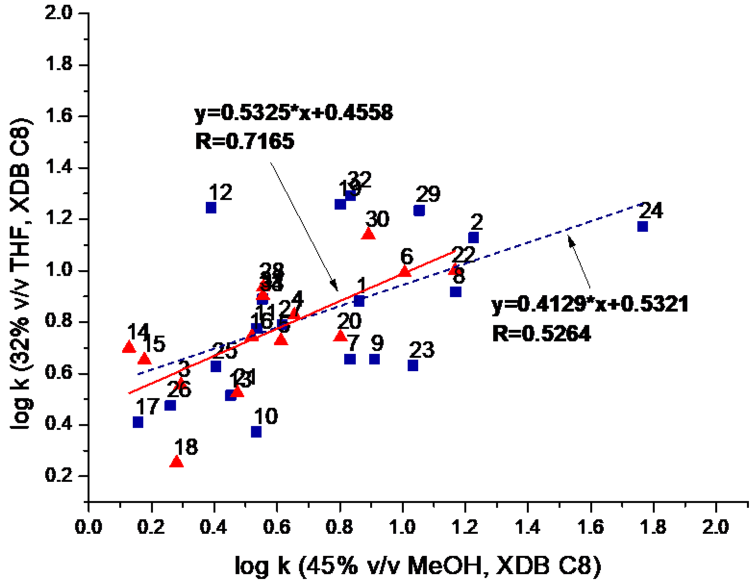

Figure S16. Log k in 32% v/v THF plotted against log k in 45% v/v MeOH. XDB C8 stationary phase. Solute numbers as in Table 2.

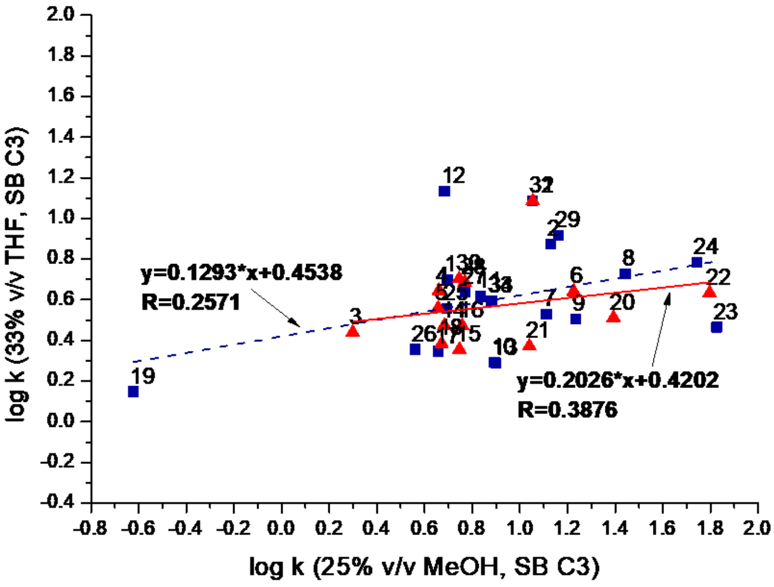

Figure S17. Log k in 33% v/v THF plotted against log k in 25% v/v MeOH. SB C3 stationary phase. Solute numbers as in Table 2.

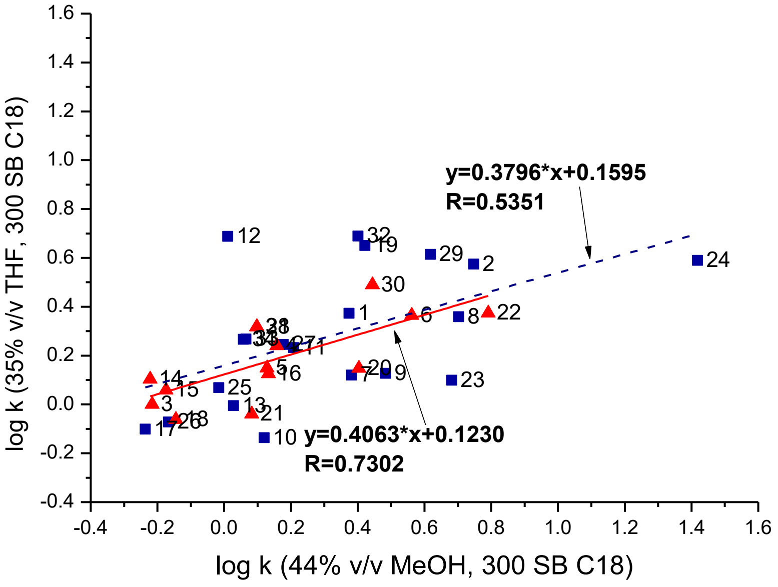

Figure S18. Log k in 35% v/v THF plotted against log k in 44% v/v MeOH. 300 SB C18 stationary phase. Solute numbers as in Table 2.

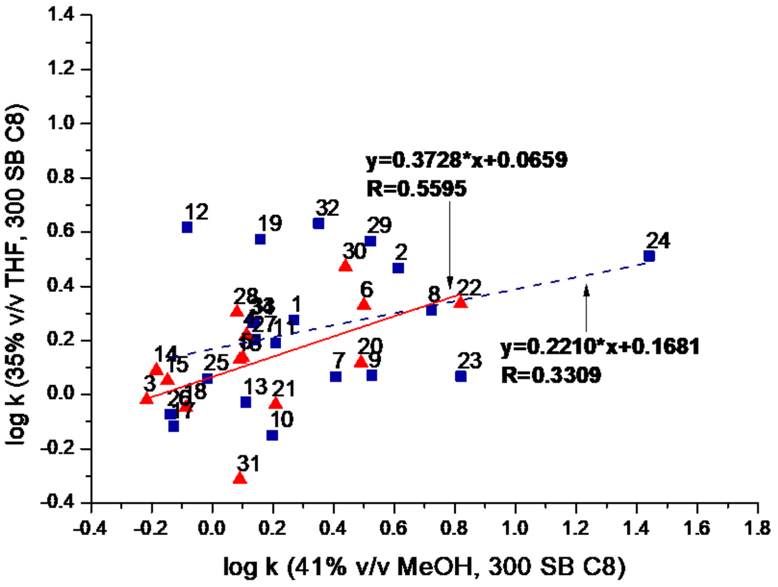

Figure S19. Log k in 35% v/v THF plotted against log k in 41% v/v MeOH. 300 SB C8 stationary phase. Solute numbers as in Table 2.

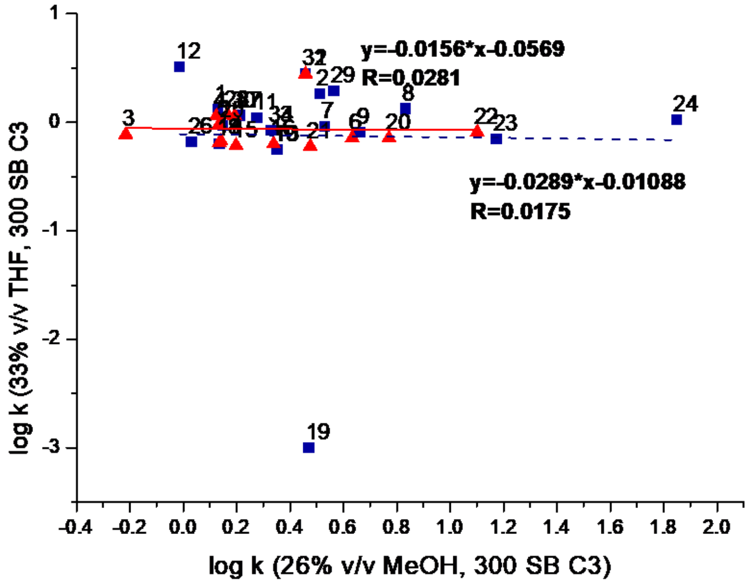

Figure S20. Log k in 33% v/v ACN plotted against log k in 26% v/v MeOH. 300 SB C3 stationary phase. Solute numbers as in Table 2.

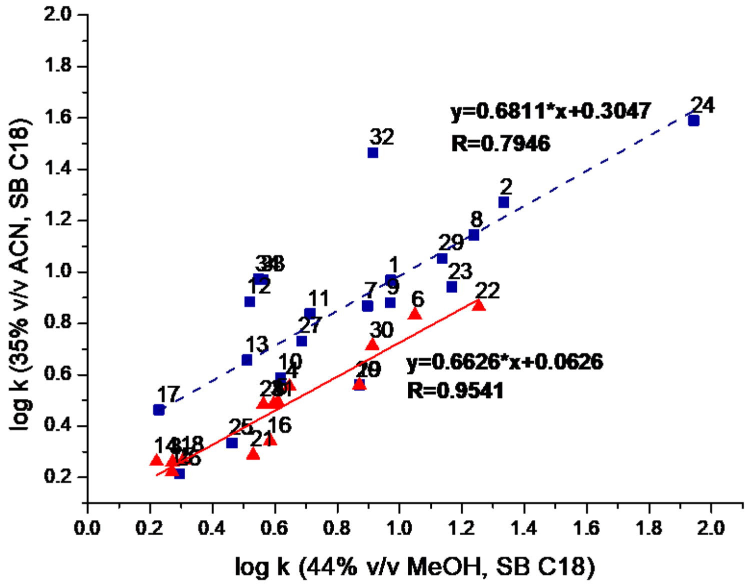

Figure S21. Log k in 35% v/v ACN plotted against log k in 44% v/v MeOH. SB C18 stationary phase. Solute numbers as in Table 2.

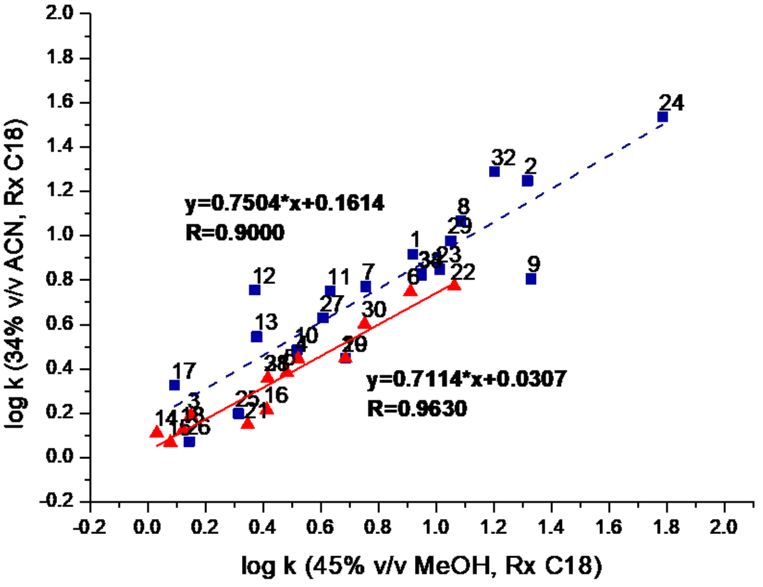

Figure S22. Log k in 34% v/v ACN plotted against log k in 45% v/v MeOH. Rx C18 stationary phase. Solute numbers as in Table 2.

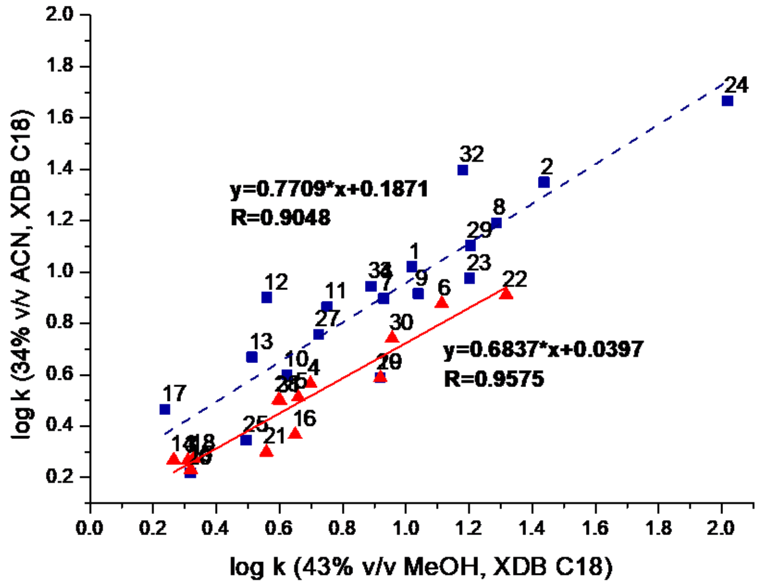

Figure S23. Log k in 34% v/v ACN plotted against log k in 43% v/v MeOH. Eclipse XDB C18 stationary phase. Solute numbers as in Table 2.

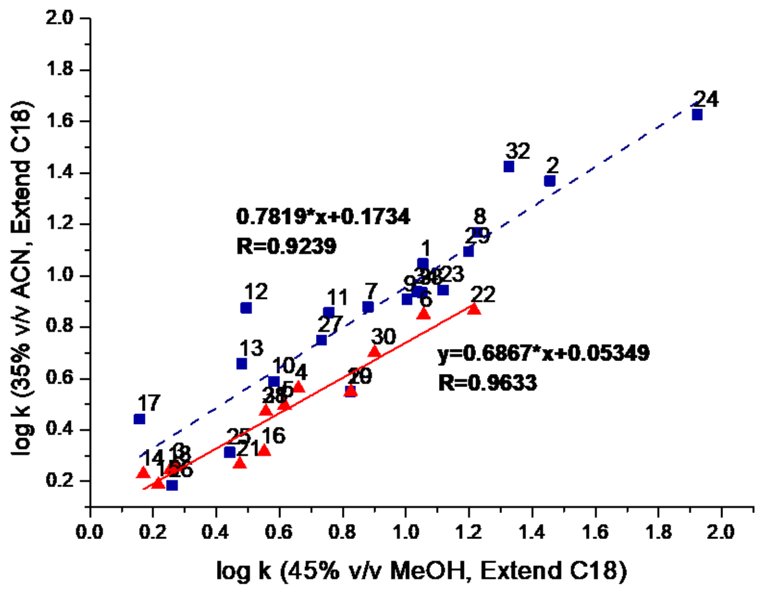

Figure S24. Log k in 35% v/v ACN plotted against log k in 45% v/v MeOH. Extend C18 stationary phase. Solute numbers as in Table 2.

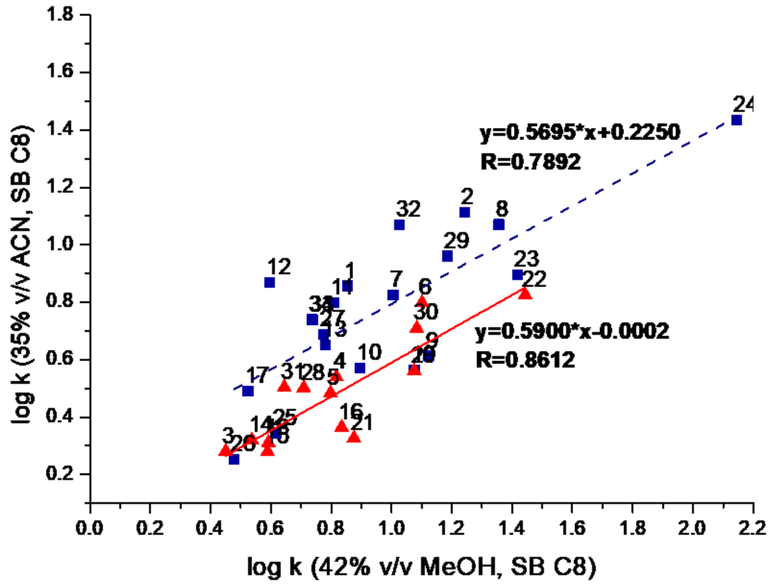

Figure S25. Log k in 35% v/v ACN plotted against log k in 42% v/v MeOH. SB C8 stationary phase. Solute numbers as in Table 2.

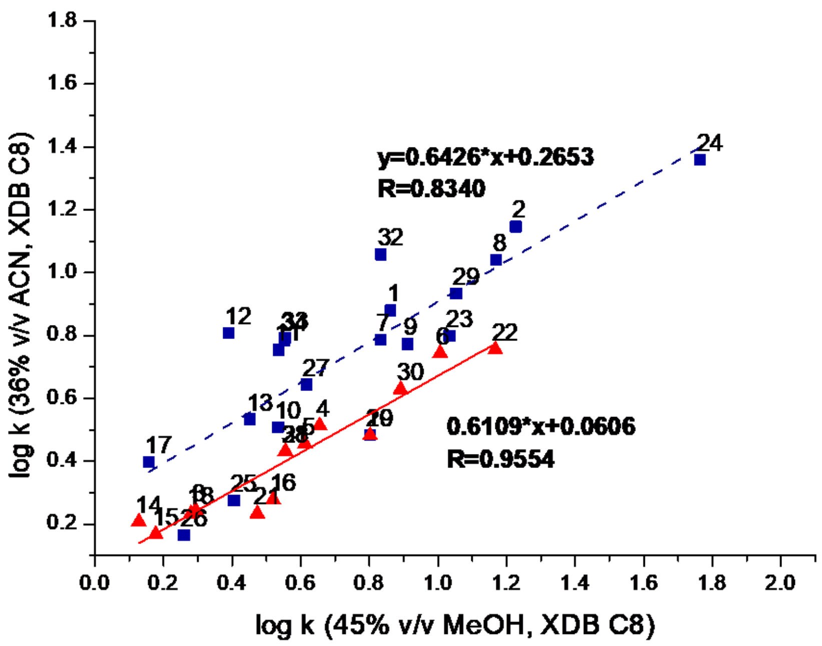

Figure S26. Log k in 36% v/v ACN plotted against log k in 45% v/v MeOH. Eclipse XDB C8 stationary phase. Solute numbers as in Table 2.

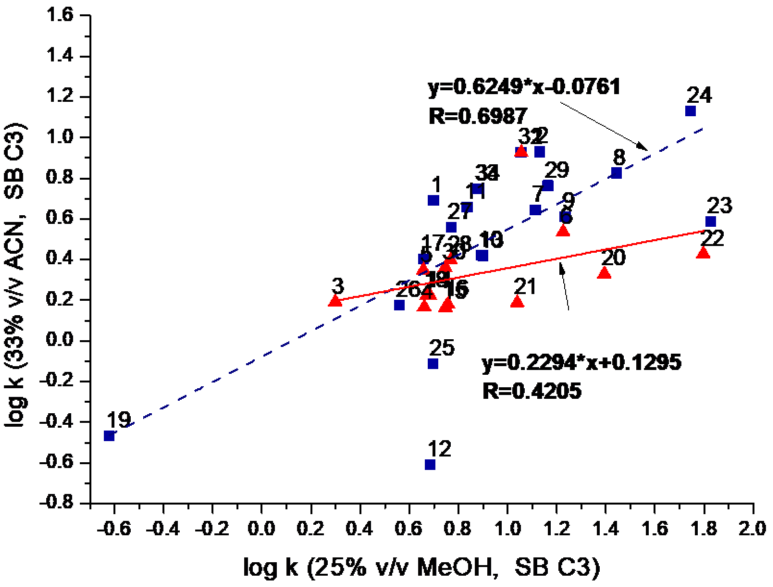

Figure S27. Log k in 33% v/v ACN plotted against log k in 25% v/v MeOH. SB C3 stationary phase. Solute numbers as in Table 2.

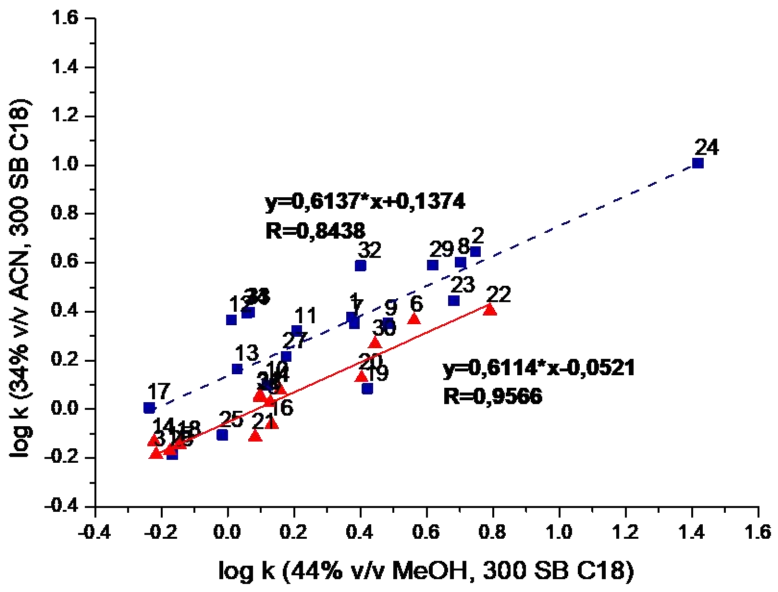

Figure S28. Log k in 34% v/v ACN plotted against log k in 44% v/v MeOH. 300 SB C18 stationary phase. Solute numbers as in Table 2.

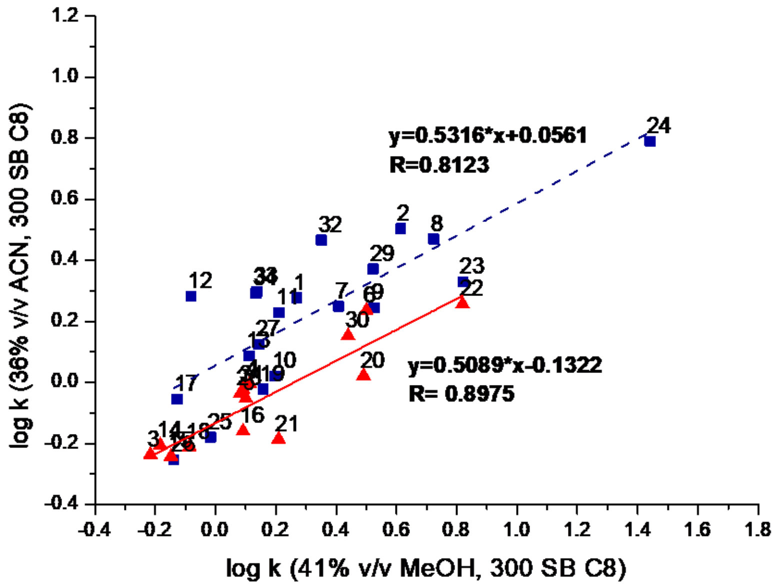

Figure S29. Log k in 36% v/v ACN plotted against log k in 41% v/v MeOH. 300 SB C8 stationary phase. Solute numbers as in Table 2.

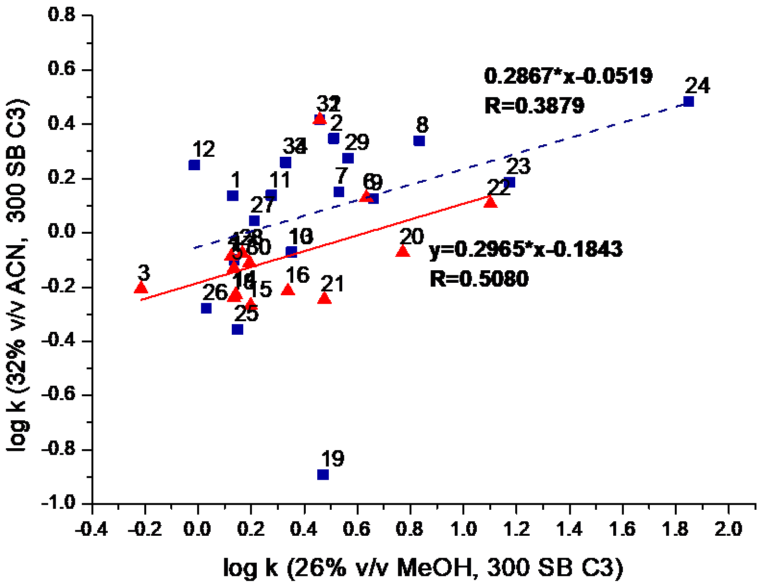

Figure S30. Log k in 32% v/v ACN plotted against log k in 26% v/v MeOH. 300 SB C3 stationary phase. Solute numbers as in Table 2.

Table 1. Structural formulas of substances used in experiments.

Lp.	Substance	Structural formula
1	Benzene	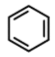
2	Toluene	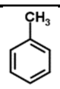
3	Phenol	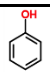
4	o-Cresol	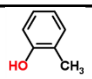
5	p-Cresol	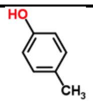
6	2-Naphtol	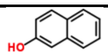
7	Methyl phenylacetate	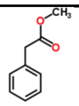
8	Ethyl phenylacetate	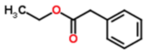

**9**	Methyl benzoate	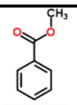
**10**	Acetophenone	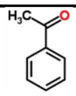
**11**	Nitrobenzene	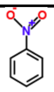
**12**	1,3,5-Trinitrobenzene	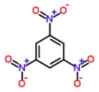
**13**	Benzonitrile	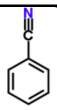
**14**	1,5-Dihydroksynaphtalene	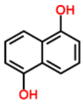
**15**	1,6-Dihydroksynaphtalene	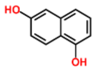
**16**	1,7-Dihydroksynaphtalene	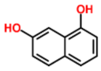
**17**	4-Cyanobenzaldehyde	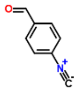
**18**	2-Cyanophenol	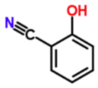
**19**	Dimethyl-4,4’-diphenyl dicarboxylate	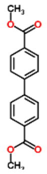
**20**	Ethyl 4-hydroxybenzoate	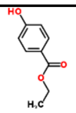

**21**	Methyl 4-hydroxybenzoate	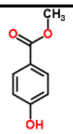
**22**	Propyl 4-hydroxybenzoate	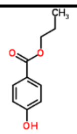
**23**	Dimethyl isophthalate	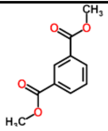
**24**	Diethyl terephthalate	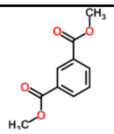
**25**	4-Nitrobenzaldehyde	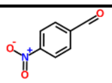
**26**	4-Nitrobenzyl alcohol	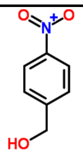
**27**	2-Nitrophenol	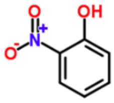
**28**	3-Nitrophenol	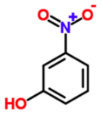
**29**	2-Nitro-4-chlorophenol	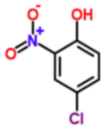
**30**	2-Metylo-4-nitrophenol	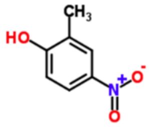
**31**	4-Nitrophenol	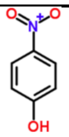

**32**	1-Chloro-2,4-dinitrobenzene	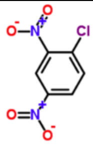
**33**	1,2-Dinitrobenzene	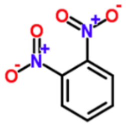
**34**	1,4-Dinitrobenzene	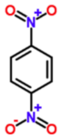
